# Community participation for malaria elimination in Tafea Province, Vanuatu: Part I. Maintaining motivation for prevention practices in the context of disappearing disease

**DOI:** 10.1186/1475-2875-9-93

**Published:** 2010-04-12

**Authors:** Jo-An M Atkinson, Lisa Fitzgerald, Hilson Toaliu, George Taleo, Anna Tynan, Maxine Whittaker, Ian Riley, Andrew Vallely

**Affiliations:** 1 Pacific Malaria Initiative Support Centre, Australian Centre for International and Tropical Health, School of Population Health, University of Queensland, Brisbane, Australia; 2 Save the Children, Port Vila, Vanuatu; 3 National Vector Borne Disease Control Programme, Ministry of Health, Port Vila, Vanuatu

## Abstract

**Background:**

In the 1990s, the experience of eliminating malaria from Aneityum Island, Vanuatu is often given as evidence for the potential to eliminate malaria in the south-west Pacific. This experience, however, cannot provide a blueprint for larger islands that represent more complex social and environmental contexts. Community support was a key contributor to success in Aneityum. In the context of disappearing disease, obtaining and maintaining community participation in strategies to eliminate malaria in the rest of Tafea Province, Vanuatu will be significantly more challenging.

**Method:**

Nine focus group discussions (FGDs), 12 key informant interviews (KIIs), three transect walks and seven participatory workshops were carried out in three villages across Tanna Island to investigate community perceptions and practices relating to malaria prevention (particularly relating to bed nets); influences on these practices including how malaria is contextualized within community health and disease priorities; and effective avenues for channelling health information.

**Results:**

The primary protection method identified by participants was the use of bed nets, however, the frequency and motivation for their use differed between study villages on the basis of the perceived presence of malaria. Village, household and personal cleanliness were identified by participants as important for protection against malaria. Barriers and influences on bed net use included cultural beliefs and practices, travel, gender roles, seasonality of mosquito nuisance and risk perception. Health care workers and church leaders were reported to have greatest influence on malaria prevention practices. Participants preferred receiving health information through visiting community health promotion teams, health workers, church leaders and village chiefs.

**Conclusion:**

In low malaria transmission settings, a package for augmenting social capital and sustaining community participation for elimination will be essential and includes: 'sentinel sites' for qualitative monitoring of evolving local socio-cultural, behavioural and practical issues that impact malaria prevention and treatment; mobilizing social networks; intersectoral collaboration; integration of malaria interventions with activities addressing other community health and disease priorities; and targeted implementation of locally appropriate, multi-level, media campaigns that sustain motivation for community participation in malaria elimination.

## Background

With significant financial and technical support being made available through The Global Fund to fight AIDS, Tuberculosis and Malaria ('The Global Fund') and the AusAID Pacific Malaria Initiative, the Vanuatu and Solomon Islands governments are embarking on an ambitious long-term endeavour to eliminate malaria from their island nations. The successful elimination of malaria from Aneityum Island, Tafea Province, Vanuatu, in the mid-1990s has given hope that a coordinated and sustained effort to eliminate malaria in Vanuatu and Solomon Islands is achievable.

The experience of eliminating malaria from Aneityum will be invaluable to eliminating malaria in Tafea province generally, but cannot necessarily provide a blueprint for the larger islands. Tanna for example, a larger island of Tafea with a current population of around 25,000 inhabitants [1], represents a much more complex social and environmental context than Aneityum, which had a population of 718 individuals in three principal community groupings when elimination was achieved [2,3].

A key component of the elimination strategy for Tafea Province is malaria prevention through universal coverage and year-round use of long-lasting insecticidal nets (LLINs) of which distribution commenced in 2008 (as described in the National Malaria Action Plan 2008 - 2011). The previous distribution of conventional insecticide-treated bed nets (ITNs) in Tafea Province contributed to the incidence of malaria decreasing significantly and it is now at a comparatively low level. A schools-based parasitology survey conducted among 5,300 children aged 2-10 years by the Vector Borne Disease Control Program (VBDCP), Ministry of Health (MoH) with support from the Pacific Malaria Initiative Support Centre (PacMISC) in mid-2008 revealed an overall malaria prevalence on Tanna of 1.0% for *Plasmodium falciparum *(accounting for 32% of infections) and 2.2% for *Plasmodium vivax *(accounting for 68% of infections) [4]. Using Bayesian geostatistics, the predicted spatial distribution of malaria on Tanna has been mapped and found to be similar for *P. vivax *and *P. falciparum *malaria with a heterogeneous distribution and 'hotspot' foci in coastal areas in the north, south-east and around the capital Lenakel in the west [4].

One of the potential consequences of malaria having been reduced to such low levels and with remaining transmission occurring in defined foci is that it can be perceived as having a relatively low priority by health workers and communities, particularly in areas where the disease is rarely seen. This may cause prevention practices in Tafea Province to become abandoned, and coupled with intra- and inter-island population movement, may result in significant malaria resurgence [5]. Yaws on Tanna provides a notable example of the consequence of local-level neglect of a low prevalence disease. Yaws was one of the first diseases targeted for global eradication in the 1950s. Once a 95% global reduction of the disease occurred vertical programmes of many countries were dismantled and eradication activities for the 'last cases' were integrated into primary care systems. In Vanuatu, initial success reduced infection from 7.3% to 0.86%. However, yaws re-emerged on Tanna in the 1980s as a result of a lack of resources, isolation, community resistance and waning attention and commitment to eradication activities [6]. Despite a mass screening and treatment programme in 1989, which achieved 91% coverage, a survey in 2007 among adults and children reported the seroprevalence of yaws on Tanna to be 17% [7].

Important research has recently been carried out to better understand the local technical elements of malaria and its transmission in Tafea Province, to assist with planning for malaria elimination. This has included entomology, parasitology and serology surveys as well as malaria risk mapping of Tanna Island [4,8]. Technical elements such as these provide essential guidance to the elimination programme. Equally important is an understanding of local perceptions, priorities and social mechanisms that impact on health behaviours and can moderate the success of infectious disease control and elimination programmes [9].

The Aneityum experience highlights the importance of community participation in achieving and sustaining malaria elimination [3]. With disappearing disease and a more complex social and environmental context, obtaining and maintaining community enthusiasm and participation in strategies to eliminate malaria in the rest of Tafea Province will be significantly more challenging. Involving communities in strategy planning and implementation as well as engaging and supporting social networks in programme delivery will assist in building social capital that will be vital to maintaining community participation and motivation despite low levels of disease [10].

Previously reported barriers to bed net use have included side effects, fear of the insecticide and reduced circulation of air onto the sleeping person, while motivators have been fear of malaria and mosquito nuisance [11,12]. In the context of disappearing malaria, motivation to use LLINs may become increasingly dependent on the inconsistent variable of mosquito nuisance, which could jeopardize the malaria elimination programme. Malaria elimination in Tafea Province will require very high levels, if not complete, bed net coverage and use, therefore a detailed understanding of the factors that influence their use and acceptability is essential.

This research was undertaken as part of an initial scoping mission to inform the community engagement and participation strategy and the development and dissemination of IEC (information, education and communication) materials in Tafea Province to support the malaria elimination programme. The results presented here relate to community perceptions and practices for malaria prevention (particularly bed net acceptability and use); influences on these practices including risk perception, key social actors and how malaria is contextualized within broader community health and disease priorities; and the most effective avenues for channelling health information on Tanna. An analysis of community understanding of malaria, its symptoms, causation and how these factors influence treatment-seeking behaviours will be described separately.

## Method

### Study area and target population

This study was carried out on Tanna Island, Tafea Province in southern Vanuatu July to August 2009. Tanna Island consists of tropical forest, grassy plains, a 1,000-metre high mountain range and a well-populated central plateau (Middle Bush). There are five indigenous languages spoken on Tanna with the prominent language being Bislama. The socio-political landscape is hierarchically organized on the basis of sex and age. Although egalitarianism governs social interaction between adult men, village chiefs and church leaders enjoy substantially more influence and prestige [2].

The Tannese are primarily engaged in subsistence farming, producing staples such as taro and yams as well as a range of fruit and vegetables. They also plant cash crops such as coconuts and coffee. The average annual cash income is less than $550 AUD per family [2]. The division of labour between men and women on Tanna is somewhat muted. Although men do much of the heavy work, and women are primarily responsible for domestic duties, both sexes contribute to working in vegetable gardens, cooking and caring for children [2].

Joint British and French colonial rule in Vanuatu in the early 1900s resulted in conversion of two-thirds of the population of Tanna to Christianity of which there remains numerous active denominations. In reaction to foreign rule a number of social movements arose from the late 1930s, which encouraged a return to traditionalist or *Kastom *practices. The best known of these on Tanna is the John Frum movement that remains an important religious and political group today [2]. A prominent cultural feature of each hamlet (cluster of villages) is the *Nakamal*, which is a place primarily where men convene to drink kava (a sedating drink made from the roots of the *Piper methysticum *plant) but also a place where people meet to dance, exchange goods and resolve disputes. There are many hundreds of these *Nakamals *across Tanna [2].

The study areas were purposefully selected to capture the views of those living in high and low malaria transmission risk areas as identified in a parasitology survey carried out in Tanna in 2008 by the VBDCP, MoH [4]. In addition, these areas were selected to take into account potential differences in community attitudes and perceptions across the island. Therefore, study activities were carried out in North Tanna, Middle Bush and South Tanna. Specific villages within those areas were purposefully selected in consultation with local stakeholders (which included chiefs, church leaders, local government officials, health workers, teachers, women's and youth group representatives) in order to ensure a breadth and depth of insights. Field researchers with village leaders nominated men, women and youth for invitation to participate in the research activities (aiming to capture a variety of views in the community). Village leaders also assisted in identifying key community informants to be approached for interview.

### Procedure

For data triangulation a variety of qualitative and participatory tools were used in each of the three villages including Focus Group Discussions (FGDs), Key Informant Interviews (KIIs) and informal observation (field journal kept by supervising field researcher). In addition, workshops were carried out using a number of participatory methods such as community mapping of malaria risk factors in each village as well as listing and ranking exercises to determine health and disease priorities and preferences for receiving health information. Separate FGDs and participatory workshops were carried out with men, women and youth (mixed gender) groups in order to facilitate open, constructive dialogue. Transect walks were also carried out in each village in which researchers accompanied by community members observed and documented similarities and differences in bed net use between households.

Two male and two female research officers were recruited locally to carry out this research and were supervised in the field by a researcher from the School of Population Health, University of Queensland (SPH, UQ). The field research team were provided training in conducting FGDs, participatory workshops, KIIs and transect walks as well as data management and research logistics by a collaborative team from SPH, UQ and Save the Children Australia (SCA) which included an experienced social scientist.

Semi-structured discussion guides were used to direct FGDs, KIIs and participatory workshop activities. Research activities were carried out primarily in Bislama and on occasion in the local area dialects. The two male field researchers were Tannese and were fluent in some of the local dialects. Where this was not the case, local facilitators were used to assist with translation.

### Data analysis

Digital recordings of the FGDs and KIIs were taken and directly transcribed and translated from Bislama (or local dialect) to English by the field research team. This data was triangulated with reports generated immediately following each participatory workshop, transect walk and with informal observations documented in the field journal. Data was coded around the main topics of the interview/workshop guides and entered into NVivo 8 software (QSR International Pty Ltd, Australia). The principal investigator subjected the data to thematic analysis, organising data into identifiable themes and patterns of behaviour [13]. Areas of consensus and divergence were identified and a 'realist method' was used to understand participants' realities, experiences and meanings. This approach has previously been reported to be appropriate for working within a participatory paradigm particularly where research findings are informing policy development [13].

### Ethical aspects

This research was approved by the Vanuatu Ethics Committee, Ministry of Health, Vanuatu and the Behavioural & Social Sciences Ethical Review Committee, University of Queensland, Australia. Volunteers were provided with a written participant information sheet. The purpose of the FGD/participatory workshop/KII was explained to them by a field officer. Participants were then asked to provide informed consent (written or witnessed thumbprint for FGDs and key informant interviews, verbal consent for participatory workshops) prior to commencing the research activity. All FGDs and KIIs were recorded using a digital tape recorder. Participants of the FGDs and participatory workshops were provided light refreshments in recognition of their valuable contribution to this research. For each FGD and participatory workshop basic demographic data of participants was recorded including age, education and religious affiliation.

## Results

In total, nine FGDs, seven participatory workshops, 12 KIIs and three transect walks were carried out in three villages across Tanna. A field journal was kept of informal observations made by the supervising field researcher during the village stays. The number of participants in each FGD ranged from six to 13 and participatory workshops had between seven and 30 participants. Background noise rendered the Middle Bush primary caregiver FGD recording inaudible, however, information could be retrieved from comprehensive reports written by the research team following each FGD.

Three additional FGDs and two participatory workshops were planned but were unable to be carried out due to festival activities and time limitations during the village visits. Although participants were primarily from the three villages visited, a small number from neighbouring villages also participated in the research activities. Participants represented a variety of beliefs systems, primarily those of Christian denominations, but also included individuals identifying with *Kastom *beliefs such as the John Frum movement (Table 1) who are known to reject many modern practices. The age range of female participants was 20 - 70 years, males 24 - 57 years and youth 15 - 34 years. Youth were defined by the community as young unmarried adults. The proportion of female participants with primary or secondary education was 57.1% compared to males at 77.3% and youth at 100% (Table 2).

**Table 1:** General religious affiliations of participants by study village.

Location of study village	Faith-based groups represented	Percentage of participants
**North Tanna**	Christian denominations (5)*	82%
	
	*Kastom *(incl. John Frum)	14%
	
	Unspecified	4%

**Middle Bush**	Christian denominations (6)*	54%
	
	*Kastom *(incl. John Frum)	23%
	
	Other (Baha'i, independent religion)	23%

**South Tanna**	Christian denominations (4)*	100%

*Denotes number of different Christian denominations

**Table 2:** Level of education of participants by gender and study village.

Location of study village	Level of education	Percentage of men	Percentage of women	Percentage of youth
**North Tanna**	None	-	45.5	Not available
	
	Primary	66.7	54.5	Not available
	
	Secondary	33.3	-	Not available

**Middle Bush**	None	44.4	53.8	-
	
	Primary	44.4	15.4	39.5
	
	Secondary	11.1	30.8	60.5

**South Tanna**	None	14.3	27.3	-
	
	Primary	85.7	63.6	27.3
	
	Secondary	-	9.1	72.7

**Over all study villages**	None	22.7	42.9	-
	
	Primary	63.6	42.9	37.0
	
	Secondary	13.7	14.2	63.0

The study villages across Tanna were generally homogeneous with regards to the key issues being investigated in this research (Table 3) except for the frequency and motivation for bed net use, where the Middle Bush village differed in their responses compared to the North and South Tanna villages, discussed in detail below. Where participant responses differed between study villages, gender or participant groups they are highlighted in the results presented, otherwise similar responses have been reported collectively.

**Table 3:** Comparison of study villages by key outcomes

Location of study village	Types of prevention practices reported	Frequency & motivation for bed net use	Influences on preventative health behaviours	Community suggestions for participation in malaria elimination
**North Tanna**	Use of bed nets Clean home/personal hygiene Protective clothing Tidy village/source Insect spray	Some reported seasonal use others reported year round use (varies at household & individual levels) Motivation to nets for both mosquito nuisance and protection from malaria Net rarely used when travelling	Health officers most influential (VHW, nurse) Church leaders, chiefs & Village elders Teachers Mothers most influential on health behaviours of children Risk perception Acceptability and Practical issues	Take advice of village leaders Set example for others Tidy village/source reducton activities Use bed nets Mosquito spray and coils Use fire/smoke for protection outside Improve roads to Lenakel/ closer clinics for treatment

**Middle Bush**	Use of bed nets Clean home Tidy village/source reduction Drinking clean water Blanket for protection when sleeping during travel	Mostly seasonal use of bed nets Primary motivation is mosquito nuiscance Net rarely used when travelling	Doctors & health officers Cheif & church leaders Teachers Household heads influence family practices Mothers influence health practices of children Risk perception Acceptability and practical issues	Village leaders to spread messages Leaders to set example for others Tidy village/source reduction activities Use bed nets Improve access to early and effective treatment

**South Tanna**	Use of bed nets Clean home/personal hygiene Tidy village/source reduction Fire/smoke as repellent Blanket for protection when travelling	Some reported seasonal use others year round use (varies at household & individual levels) Motivation to use nets for both mosquito nuiscance and protection from malaria Net rarely used when travelling	Health officers Church leaders, chief and village elders Household heads influence family Mothers influence health practices of children Risk perception Acceptability and practical issues	Village leaders to encourage participation Tidy village/source reduction activities Use bed nets Support IRS

**Homogeneity/ ** **Heterogeneity of ** **responses**	**Homogeneity in types of ** **protection methods ** **used across the 3 villages**	**Heterogeneity between ** **the Middle Bush village ** **and other 2 villages due ** **to absence of malaria**	**Homogeneity in ** **influences on health ** **behaviours across the ** **3 villages**	**Homogeneity in ideas for ** **participation in malaria ** **prevention across the ** **3 villages**

### Methods of malaria prevention used

The reported methods of protection from malaria used by adults and children did not differ. The primary protection method identified by all participant groups was the use of bed nets. Cleanliness was also frequently reported as essential for malaria prevention. This ranged from ensuring that adults and children wash daily and wear clean clothes to keeping one's house and yard clean and tidy (including some source reduction activities such as draining of collected rain water). A few participants reported the importance of making sure children do not walk outside during cold and windy times and of ensuring the consumption of clean water for the prevention of malaria. Typical of these responses was the following quote:

*'Some of the preventive measures that we use are; using mosquito nets, don't drink from dirty pools, boil water to make sure water is safe for the family to drink or cook with; and also cleaning our houses.' *(FGD, Middle Bush man)

Very few participants reported the role of witchcraft in malaria transmission; those that did discussed it in a historical context.

*'Some people mistake the symptoms of malaria and think the illness is caused by witchcraft so they take all those strong traditional medicines which sometimes cost them their lives. We don't take responsibility for protecting ourselves properly and blame other causes for the illness. Now, after a lot of information from the health workers we know that malaria is an illness caused by mosquitoes.' *(KII, South Tanna chief)

Although most participants recognized that mosquito bites can cause malaria, prevention practices relating to ensuring personal cleanliness, boiling water prior to consumption and keeping children out of the wind and cold indicate that many perceive malaria transmission to occur through additional means.

Other methods of protection reported by a few participants were the use of long clothing, a blanket, mosquito coils and the traditional practice of burning coconut husks or green leaves to create smoke for repelling mosquitoes. These methods continue to be used particularly by those travelling away from their village or camping by their gardens as an alternative to the use of bed nets.

#### Bed net coverage

Most participants reported using the LLINs distributed the previous year and many continue to use the older conventional ITNs. Transect walks confirmed that conventional ITNs continue to be used due to insufficient LLINs for coverage of all household members and were reported as being 2 - 7 years old. These older conventional ITNs ranged from being in relatively good condition to being considerably torn but were being used in the absence of available new nets. Insufficient bed nets during holiday seasons was commonly reported. Visiting family or friends may stay for periods of up to several weeks and use old nets that have been saved by householders, but there are often insufficient nets to accommodate additional guests.

#### Bed net acceptability and barriers to consistent use

Despite the common perception that mosquitoes can pass through the larger mesh spaces of the Olyset bed nets, the majority of participants over all three areas of Tanna reported relatively good acceptability of the LLINs received for the first time the previous year and described them as being strong, larger in size and having better ventilation than the previous conventional ITNs. Most participants expressed a preference for larger sized nets for better ventilation and less movement restriction. Although most participants reported the LLINs to be acceptable, there remain a number of barriers to their consistent use including travel, cultural beliefs and practices, gender roles and seasonality of mosquito nuisance.

Inter- and intra-island travel for work, holiday and festival activities was reported in all three study villages with limited effective malaria protection used during these times. Particularly, travel to and participation in Provincial festivals can result in bed net non-use for a period of several days to a week or more. For example, in July 2009 people from all over Tafea travelled to the Provincial capital (Lenakel) for a week-long arts festival which was followed by Independence Day celebrations. A number of participants reported that during this time many people slept without bed nets.

Cultural beliefs and practices were also reported as barriers to bed net use. It was commonly stated that people with strong *Kastom *beliefs were either not using bed nets as a result of their lack of acceptability or not using them consistently due to regular all night dancing rituals. Fear or dislike of the insecticide was identified on a number of occasions as the primary reason for net non-use by those with *kastom *beliefs.

*'....some don't (use nets) due to their strong belief in Kastom....they are afraid of the chemical in the net, that it might cause death to them.' *(KII, North Tanna Pastor)

There are gender-specific barriers to bed net use. The traditional practice of women retreating to separate huts during the time of menstruation still occurs in some parts of Tanna (particularly in Middle Bush). Mosquito nets are reportedly not always available for women sleeping in these menstruation huts.

*'Here in Tanna, when the mothers go through their menstruation they go and sleep in another house apart from the fathers and they may not use nets. This is a common practice in the whole of Tanna.' *(FGD, Middle Bush man)

Barriers to consistent bed net use by men on Tanna include livelihood activities such as fishing, camping by vegetable gardens, and travel for work; as well as evening social activities. Regular evening kava drinking at the *Nakamal *was commonly reported as a practice that reduces the use of bed nets amongst men.

*'...most of the men in this village do not like using the nets. They say they feel hotter when they go under the nets and (are) under the influence of kava or alcohol. Sometimes they do not sleep under their nets if they camp in their gardens and if they go out fishing.' *(KII, South Tanna chief)

Many participants over all three areas of Tanna reported seasonal bed net use only, which is primarily influenced by mosquito nuisance and evening temperature, and fear of malaria in North and South Tanna. Some typical comments were:

*'It depends on the mosquitoes, if there are more then we use the nets, but not the whole year.' *(FGD, Middle Bush youth)

*'The reason why I use a bed net is because when I go to bed and am snoring away there won't be any mosquito noise disturbing me.' *(FGD, North Tanna man)

*'During the hot seasons we don't use nets, it is only during mosquito seasons that we use nets.' *(FGD, South Tanna man)

#### Maintenance of bed nets

'Mothers' were identified by the majority of participants as being primarily responsible for washing bed nets (although some youth reported washing their own nets). The frequency of net washing varied with some reporting it occurred whenever the nets were dirty, others once or twice a year and a few reported their nets were never washed for fear of removing the insecticide.

*'If the malaria people told us that even if you wash the nets the power will still remain, then I would wash my net, but they haven't said it so I don't want to wash my net' *(FGD, South Tanna youth)

Many participants reported confusion between the washing instructions of the previous conventional ITNs (twice per year prior to retreatment with insecticide and hang to dry in a shady area) and the newer Olyset LLINs (washing every couple of months followed by regeneration of insecticide by placing the net in a plastic bag in direct sunlight). Some participants reported to be awaiting the arrival of the malaria team for re-treatment of their LLINs prior to washing them. None of the participants interviewed reported using the correct procedure for regeneration of insecticide in the Olyset LLINs.

### Influences on preventative health behaviours

Responses relating to who are most influential in changing preventative health behaviours in the community were similar in all three areas of Tanna and between men and women. Participants appeared to be influenced by those that they saw as an authority when it comes to health issues and by those well-known and respected in the community. Doctors, village-based health workers (which included both nurses and aid post volunteers) and visiting malaria officers were most often reported to have the greatest influence on malaria prevention practices such as bed net use. Many participants also reported church leaders as particularly influential.

Village chiefs, elders and teachers were also reported by many as having some influence over health practices. Within households it was often reported by both male and female participants that the 'mothers' are responsible for looking after the home and health of the family and were the most significant influencers on the health behaviours, particularly of children. However, some men reported that they themselves were ultimately responsible for decision making regarding bed net use in the household.

*'The father is the head of the house so he is the one that tells the children and the mother to use nets to protect them from malaria.' *(FGD, South Tanna man)

*'...we heard from the doctor that we should use the net to sleep under and so we, the fathers, as household heads, we make sure all the members of our family sleep under nets.' *(FGD, Middle Bush man)

Despite these influences the majority of participants (including youth) reported that they made their own decisions regarding malaria prevention practices such as bed net use. In addition to the influence of health officers and other community leaders, practical factors and acceptability issues (as discussed previously) as well as risk perception play a role in decision-making processes.

Perception of risk plays a significant role in influencing preventative practices such as bed net use. Several participants in each of the FGDs in North and South Tanna reported having their bed nets hanging in their household throughout the year and stated fear of malaria as their reason for doing so. Their views are summarized in the following two quotes:

*'Yes. People do sleep under bed nets because we've had people dying of malaria and (therefore) some of our people make it a habit to sleep under bed nets.' *(KII, North Tanna chief)

*'As a household head, I am afraid of the illness and I make sure my family uses nets at night.' *(FGD, South Tanna man)

**Figure 1:**
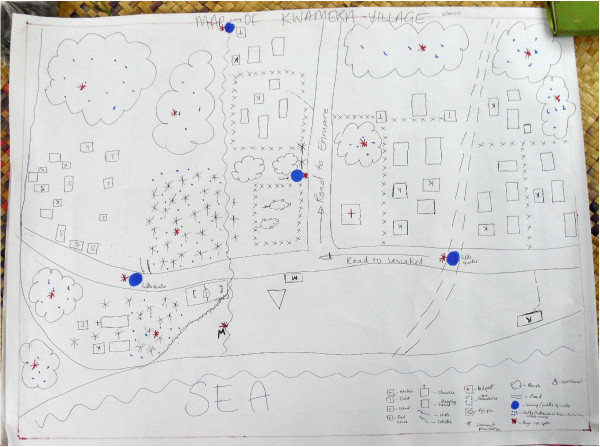
**Map drawn by South Tanna women of malaria risk factors in their village**.

Participatory mapping highlighted participants' intimate knowledge of the local environment and their ability to identify risk factors such as mosquito breeding sites and households where malaria is present (Figure 1). In addition, an exploration of health and disease priorities, and the motivators and barriers to bed net use, revealed a number of factors related to risk perception that influenced both the decision to use bed nets, and the pattern of their use. Participants that perceived themselves or their children as being at risk of malaria were more likely to use bed nets. Commonly reported factors that contributed to heightened risk perception and hence bed net use were: knowledge of malaria causation and the presence of mosquitoes; perception of malaria as a severe illness (particularly in children); presence of the disease; or recent memory of severe illness or death from malaria in the community.

*'Malaria is more severe in children than adults so they need to use bed nets every day.' *(FGD, North Tanna woman)

*'We experienced a great disaster in 1996 with malaria where everybody was sick, the adults and the children. The adults were lucky because they were strong and they can tolerate the illness, whereas the children were not very strong and they sometimes fell unconscious because the fever was very strong in them....everybody has seen the effect of malaria in their lives....and see the importance of sleeping under nets to protect themselves from malaria.' *(KII, South Tanna youth leader)

Commonly reported factors that contributed to a perception of reduced risk of malaria and hence bed net non-use were: inaccurate understanding of malaria causation, if the perceived risk of the insecticide in the net was greater than that of contracting malaria, a lack of mosquito nuisance or an absence of the disease in the community.

*'People don't get sick (with malaria) even when they don't sleep under nets so they might not want to use bed nets.' *(FGD, Middle Bush youth)

*'The insecticides can make people sick if they breathe in or not wash their hands properly.' *(FGD, Middle Bush woman)

Community health and disease priorities were obtained through consensus among participants in each of the participatory workshops (Table 4), and as well as influencing risk perception, were found to play a role in determining community motivation for participation in malaria prevention. The priority accorded malaria in study communities was established on the basis of how common and how severe it was perceived to be relative to other health issues experienced. Despite low transmission, malaria is considered a high priority in both North and South Tanna study villages (particularly in children) because it is still regarded as a current and serious threat. Malaria was not considered a high priority in Middle Bush however, except among youth participants. This is due to it not being considered a common illness in the area in recent times.

**Table 4:** Community health and disease priorities for adults & children determined through listing/ranking activities during PLA workshops*

	North Tanna village	Middle Bush village	South Tanna village
**Men** (Adult health & disease priorities)	Not available	Back ache Cold/flu Stomach ache Arthritis Ulcers	High blood pressure Stroke Malaria Cancer Back ache Leprosy

**Women** (Adult health and disease priorities	Prolapsed uterus Malaria Back ache TB Head ache	Prolapsed uterus Cancer TB Back ache Long periods	Head ache Chest pain Malaria Long periods Back ache Diarrhoea

**Youth** (Adult health & disease priotities)	Not available	Cancer TB High blood pressure Malaria Ulcers	Malaria High blood pressure Diabetes

**Children** (Health & disease priorities based on consolidated listing/ranking activities by men, women & youth during PLA workshops)	Malaria Headache Asthma Measles Fever	Diarrhoea Fever Ulcers Scabies Cough	Malaria Fever Diarrhoea

*Listed in order of priority

Despite malaria's lack of priority in Middle Bush, motivation for participation in prevention practices is maintained by some participants as a result of risk perception that is being maintained by past experiences of severe illness or death or due to their understanding of the potential for malaria transmission as a consequence of local environmental factors that support mosquito breeding.

*'Yes here we think malaria is a big problem...we live in the rainforest part of the island and our weather is very cold and humid and it is common to have a lot of rainfall so we have a lot of mosquitoes.' *(FGD, Middle Bush man)

*'...we have had some experiences here in the past where some children were normal at birth but after becoming sick with malaria, they ended up having some disabilities.' *(FGD, Middle Bush man)

Although this risk perception is maintaining bed net use by some, seasonal mosquito nuisance is the predominant motivator for bed net use among participants in this community.

### Avenues for receiving health information

**Figure 2:**
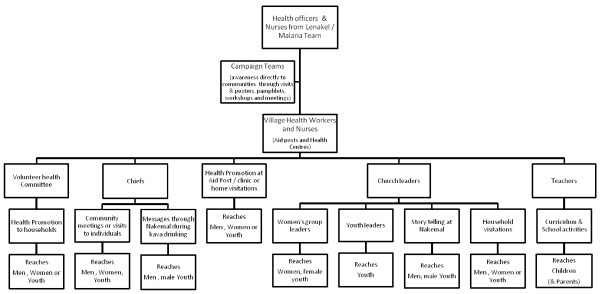
**Flow of health information on Tanna Island, Vanuatu**.

Based on participant responses across the three study villages, the current flow of health information and method of delivery is mapped out in Figure 2. Avenues for health message delivery are consistent with those people participants reported as being most influential with regards to preventative health behaviours (discussed previously). The top four preferred avenues for receiving health information, consistent across the island were through visiting health awareness teams from Lenakel, through health workers based in health centres and aid posts, through their church leaders and by the chief through their *Nakamal*.

## Discussion

Adherence to malaria prevention practices in Tanna, such as bed net use, appears to be a complex interaction between risk perception, intervention acceptability, socio-cultural factors and practical issues. These influences on preventative health behaviours have been described in the literature previously [14-18]. Despite the low prevalence of malaria on Tanna [4], its perceived priority in communities of North and South Tanna is sustained by recent memories of it being a common and severe illness to which children are particularly vulnerable. With limited transmission, low priority and comparatively lower perceived risk of malaria in the Middle Bush community, motivation for malaria prevention practices such as bed net use are being maintained primarily by mosquito nuisance. As mosquito nuisance was reported as seasonal in the three villages of this study, this motivation alone may be insufficient to achieve year round, high bed net coverage that will be required for malaria elimination. In addition, the limited use of bed nets among those with *Kastom *beliefs is of particular concern.

In such contexts, health education initiatives that attempt to elicit participation only through increasing malaria knowledge and by encouraging individuals to take responsibility for their own health will be ultimately ineffective [19,20]. Mobilising or augmenting social capital has been identified as an important prerequisite to enhancing health promoting behaviours and will be more effective than an approach that emphasizes individual rational choice [10]. Social capital, described as an overarching concept that pulls together previously described phenomena such as 'sense of community,' 'community competence,' 'collective efficacy,' 'critical consciousness,' and 'empowerment,' is thought to be critical for achieving and sustaining community participation in health programmes [10,21]. In Tanna, where existing social mechanisms appear to promote a strong 'sense of community,' and influence individual and household level decision making, complementary strategies should be employed that concurrently augment social capital, improve individual knowledge regarding malaria and it's transmission and dispel rumours and misconceptions that can create community resistance to participation in malaria prevention practices. A multi-faceted, multi-level approach will be particularly important for a programme of elimination in areas where malaria is not a perceived risk or a community priority, and where religious belief systems discourage participation.

Lessons from the Aneityum experience revealed that in order to maintain elimination once malaria is no longer considered a public health concern and external funding is withdrawn from a weak economy, communities need to internalize key issues, take ownership for programme implementation and maintenance, and have sufficient social capital to maintain momentum for the long-term [22]. The social and behavioural data obtained through this research will inform the development of a comprehensive strategy for communication and community participation in malaria elimination in Tafea Province [19]. To replicate the success of Aneityum on larger islands with more complex socio-cultural and environmental contexts, this research suggests that the strategy should include; mobilizing social networks and community leaders through consensus building processes; pulsed media campaigns (including traditional media such as storytelling, music and community theatre) to advise communities of the efforts to eliminate malaria in the Province, to increase knowledge of malaria and to address barriers to participation; strategies to overcome gender barriers; provision of outreach to remote communities such as interpersonal communication and a system for monitoring changing community perceptions and participation [23-25].

The specific strategies outlined below are presented in the context of an existing enabling environment created by a broader commitment for malaria elimination in Vanuatu that includes significant in-country and international political, policy and financial support. Table 5 summarizes the specific interventions presented here according to whether they are attempting individual behaviour change, modification of social norms or both.

**Table 5:** Summary of proposed interventions to build and maintain community participation in malaria prevention practices (particularly the use of LLINs).

Proposed interventions	Attempting individual behaviour change	Attempting modification of social norms
Engaging and augmenting social mechanisms		×

Engaging communities by:		
Integrating malaria interventions with activities addressing other community health and disease priorities		×
Obtaining multisectoral involvement		×
Providing feedback to communities of progress towards elimination		×

Communication strategy (targeted messages)		
Increasing knowledge of malaria	×	
Modifying malaria risk perception	×	×
Modifying perceptions and misconceptions of LLINs	×	×
Modifying patterns of bed net use (and conveying importance of high community coverage)	×	×
Promoting LLIN maintenance	×	×
Addressing gender specific or lifestyle related exposure to mosquitoes/malaria		×
Addressing issue of limited LLIN use while travelling	×	×

Communication strategy (channels of communication)	×	×
Storytelling, community meetings, workshops, school curriculum, community theatre, music, special events, personal selling, mass media, promotional materials and peer-led education		

### Strategies to address barriers to community participation in malaria prevention practices in Tafea Province

#### 1. Engaging and augmenting social mechanisms

Obtaining endorsement for malaria elimination from key community members found to influence health behaviours and engaging them in the planning of community-based activities will be an essential first step towards establishing effective partnerships with communities to support local-level programme implementation, surveillance, monitoring and evaluation [26-28].

Given the influence that church leaders were reported to have with regards to health behaviours and that they are able to reach people at the household and individual levels through special interest groups and house-to-house visits; engaging church leaders in the community mobilisation strategy will be important. There were as many as eight different religious affiliations represented in each of the areas that the research was conducted, therefore, mapping the presence and reach of each religious group active on Tanna will assist in identifying the many church leaders needing to be engaged in community mobilisation for malaria elimination. In addition, particular attention needs to be paid to identifying and working with *Kastom *communities to encourage participation in the programme through existing relationships between health care workers and the leaders of these communities.

Through the use of existing social mechanisms the elimination programme can capitalize on communities' intimate knowledge of local environmental risk factors for malaria and engage them in regular, coordinated, community-wide prevention measures including source reduction, personal protection and surveillance activities [29,30]. This will be particularly important in remote communities where access to treatment for malaria is considerably more difficult and whose participation in the programme is vital to achieving and maintaining zero malaria transmission.

Village health workers in North and South Tanna reported the existence of a volunteer committee made up of members of the existing aid post committee. These volunteers were also representatives of villages more distant to the aid post and therefore assisted the village health worker in providing outreach health promotion. It is unclear from the interviews undertaken in this research how wide reaching these volunteer committees are on Tanna or how many are currently operational. They may represent an important avenue for engaging remote communities in malaria elimination by providing house-to-house interpersonal communication for intervention advocacy and, therefore, should be further investigated.

Finally, social mechanisms provide a continual community presence which through regular communication and publicity can assist the programme in addressing issues that create community resistance, maintain focus on malaria elimination in the absence of the disease and avoid the problems of waning attention and commitment experienced, as for example during the previous yaws elimination campaign in Vanuatu [6].

#### 2. Engaging communities and maintaining motivation for participation

Integrating malaria interventions with activities addressing other community health and disease priorities will assist the malaria program in fostering good will and rapport with the community. For example, mobile malaria screening or campaign teams could include a medical officer who as well as providing education and treatment for malaria, could address other community health priorities and epidemiological evidence-based health needs (e.g. childhood immunisation coverage). In addition, malaria prevention practices such as environmental management could be integrated with other disease prevention programmes, such as the 'tidy village campaign', which targets dengue and diarrhoeal diseases through collaboration between the VBDCP and Environmental Health. The incorporation of education regarding malaria causation, prevention and treatment into the school curriculum is an additional strategy that would take advantage of the influential role teachers play in the lives of children and their parents. These integrated measures will require multisectoral involvement and will importantly frame the malaria elimination programme within a primary health care approach and assist in demonstrating to communities the Ministry of Health's commitment to improving their health and well-being [31].

A further strategy for maintaining motivation in the context of disappearing disease will be to provide communities with regular updates on the progress and then maintenance of malaria elimination on Tanna. Feedback could be delivered to communities through strengthened reporting systems between the provincial malaria team and the aid posts, health clinics, schools and churches.

#### 3. Communication strategy

Standardized verbal and written education will need to be developed and disseminated widely. To improve the likelihood of achieving behaviour change, the strategy for distribution of health education should be integrated with the aforementioned strategies for engaging social mechanisms and sustaining community participation. Targeted health education initiatives have not always been effective in achieving behaviour change which, as well being a consequence of economic or institutional factors, may be a result of their failure to modify social norms, particularly if messages are attempting to address barriers to participation that are socially constructed [10,32,33].

##### Messages

Inaccurate beliefs relating to the mode of malaria transmission or the safety and efficacy of interventions could affect the community's ability to take effective preventative action. This research has highlighted the need for accurate and standardized messages regarding malaria causation, symptoms, prevention and treatment. Messages will need to address fears regarding the insecticide and confusion over maintenance of bed nets (which will be further compounded by the introduction of a new brand of LLIN to be distributed in early 2010), and should elucidate the continued risk of malaria transmission during non-peak mosquito seasons.

Building motivation for year-round use of bed nets primarily on the basis of mosquito nuisance and malaria risk, may be limited in its effectiveness for a number of reasons. Firstly, risk perception is a dynamic system that is both individually and socially constructed [34]. It is influenced by individual psychological capacity at various life stages, interactions with other individuals and their characteristics, socio-cultural contexts and environmental conditions [34]. Given the intra- and inter-community differences in risk perception that inevitably arises from this complex system, standardized messages regarding the importance of using preventative measures (such as LLIN use) to mitigate risk will alone fail to provide motivation for participation to a sufficient proportion of the population. In addition, misperceptions of malaria risk resulting from variances in mosquito nuisance (particularly in areas of low or no malaria transmission, such as Middle Bush) and inaccurate understandings of malaria causation, as reported in all study areas, negatively influence motivation for using LLINs to prevent malaria.

Similar findings have been reported elsewhere and describe the initial high acceptance and use of new bed nets during the campaign period giving way to reduced usage as perceptions of their lack of usefulness (due to additional causes of malaria) and their daily inconvenience emerge [35]. Therefore, as well as addressing inaccurate perceptions of malaria causation and risk, motivation for bed net use by households could be built on their existing understanding of the vulnerability of children to malaria as well as the additional benefits of LLINs such as allowing a good night sleep for children and reducing other household insects such as bed bugs and cockroaches. In addition, messages stating the importance of high community coverage and consistent use of LLINs for successful malaria elimination (despite the reduced presence of mosquitoes) should be conveyed, with an emphasis on encouraging their peers to participate in the struggle to be rid of malaria.

Inter- and intra-island population movement for work, holiday and festival activities provides a constant and significant threat for malaria resurgence on Tanna, particularly with reports from all three study villages of limited effective malaria protection used when travelling [36]. Communities will therefore need to be encouraged to take bed nets with them when travelling and use older serviced nets if newer nets are not available.

Although the use and maintenance of bed nets is already somewhat incorporated into women's domestic routines, this could be further encouraged and supported as well as being discussed as part of men's responsibility in running households. Net maintenance kits could accompany future LLIN distribution and the repair of serviceable older nets promoted. Older, well-maintained nets could be saved for travelling of individual family members, for household guests or for use in women's menstruation huts. Motivation for women to encourage household participation in malaria prevention practices such as bed net use may be promoted by messages of the negative impact on their ability to work and carry out household tasks when they have to care for sick family members [32].

##### Avenues for message delivery

This research identified village-based health workers (nurses and aid post volunteers) and other health officers being the most influential and preferred people for the delivery of messages relating to health practices, however, these health workers are often operating at full capacity and are unable to implement any additional health promotion and community mobilisation activities as part of a malaria elimination programme. Mapping the flow of health information on Tanna and identifying other influential community members has assisted in recognising local-level resources and additional avenues for dissemination of behaviour change communication (BCC). Multiple channels of communication are preferred in order to reinforce messages and reach a greater audience; however, an understanding of current communication channels to specific audiences as well as indigenous preferences for receiving health information will enhance the efficacy of messages and ensure the most efficient use of limited resources [28,37].

Storytelling for the transmission of health messages is a traditional practice on Tanna and has been documented elsewhere as an effective means of education as it can present both positive and negative behaviours as well as ideas and values in a simple, entertaining form [38]. Other established tools for health education on Tanna reported by participants were community meetings, workshops, school curriculum and community theatre. Additional effective tools for health education identified in the literature include promotional materials (t-shirts, caps, posters, leaflets), music, special events (competitions, programme launches) personal selling, point-of-sale health promotion and mass media (TV, newspaper, radio) [28,39]. Radio, particularly the use of mini-drama series with health themes, can be an effective educational tool and has the advantage of being able to reach remote communities or those with low literacy [23,40,41]. Although transect walks in the three study communities found many households to have radios, reception was reportedly poor. Currently being explored are alternatives such as the provision of community radios and pre-recorded standardized education programmes that can be broadcast locally.

Balanced with the importance of channelling health information through influential community members is the recognition of unequal power relations (such as between men and women) and its potentially negative impact on empowerment. A feeling of lack of control over one's health is likely to hinder health-enhancing behaviours [42]. To counter this effect, peer-led education has been suggested as a means of providing a safe environment for promoting assimilation with health prevention practices [21]. Peer education has been shown to significantly increase knowledge, modify attitudes and improve protection self-efficacy [43,44]. In Tanna, existing special interest groups (e.g. women's and youth groups, groups that support victims of domestic violence etc.) could be engaged and supported in carrying out peer-led education and the delivery of a range of targeted health messages.

### A model for sustaining community participation for malaria elimination

Formative qualitative and participatory research as described here that investigates the socio-cultural, behavioural and practical issues that influence health behaviours is valuable for identifying barriers, motivators and key issues affecting community participation for initial strategy development and programme design. However, because they investigate issues at a single time point they are rarely able to provide a comprehensive picture of the dynamic influences on health behaviours and community participation nor how they evolve following programme implementation. Regular broadly implemented research throughout the Province to investigate changing community attitudes and barriers to participation during and following malaria elimination would require significant human and material resources.

A model being considered for pilot in the SW Pacific is the establishment of 'sentinel sites' for monitoring community engagement and participation in a few purposefully selected villages. This model builds on the 'qualitative monitoring' approach used successfully in the KINET Project in southern Tanzania [28]. Local volunteers could be trained and supported to carry out iterative research using a tool kit of PLA (participatory learning and action) methods/activities. This would create an avenue for genuine community engagement and participation in the design of socially and culturally acceptable intervention implementation strategies, as well as contribution to programme evaluation and modification. The potential benefits of this approach are:

• New technologies or implementation options for existing interventions (e.g. models for larviciding/source reduction activities, IRS implementation, LLIN distribution) could be trialled and modified as required at sentinel site communities prior to large scale application in order to maximize intervention success and reduce relative costs.

• Sentinel sites can provide valuable in-depth, real-time feedback to provincial malaria staff and policy makers, allowing the programme to remain responsive to changes in community perception, misconceptions, priorities, concerns and practices. Such rapid and responsive actions will go a long way to preventing community disharmony arising from unattended concerns and misconceptions that would negatively impact participation in the elimination programme.

• They provide an avenue for constructive dialogue and the exchange of views and perspectives between communities and policy makers and demonstrate programme commitment to genuine community participation.

• Sentinel sites would provide a continuous interface for the exchange of 'exogenous' and 'indigenous' knowledge and communication [28].

• If significant concerns are raised in sentinel site communities, further investigations can be carried out in other communities to investigate these issues in more depth.

• Through the PLA approach, sentinel site communities would benefit from further enhancement of social capital, the development of 'critical consciousness' and contribute to a sense of community ownership, which will assist with maintaining motivation for malaria elimination in the context of disappearing disease.

The location of these sentinel sites for community engagement and participation should aim to capture communities that differ with regard to level of malaria transmission and those in rural or urban settings.

### Limitations of the study

This study was conducted to explore issues around malaria prevention practices (particularly bed net acceptability and use); community health and disease priorities, influences on prevention practices; and avenues for receiving health information on Tanna. Vulnerability to malaria can vary due to biological, cultural, socioeconomic, environmental and institutional factors and can influence community participation in malaria prevention practices [32,33]. This research explored the influence of risk perception (which is closely linked to vulnerability) on malaria prevention practices; however, it was beyond the scope of this operational research to investigate the influence of the broader concepts of vulnerability and resilience. Further investigation into these dynamic concepts and their impact on community participation for malaria elimination could occur as part of the work carried out at 'sentinel sites' for community engagement and participation.

As with the nature of qualitative research, the results are limited in their ability to be generalized to the wider population of Vanuatu. The authors also recognize that responses relating to bed net use may be an overestimation as they are subject to social desirability bias. In addition, the importance of malaria in responses relating to health and disease priorities may be influenced by the presence of the research team asking questions about malaria. Finally, there may be some degree of loss of nuances and depth as a result of the direct transcription and translation from Bislama to English of FGD and KII recordings by the local research officers.

## Conclusion

In summary, we propose a package for augmenting social capital and sustaining community participation in malaria elimination in low transmission settings such as Vanuatu which includes: Formative research and 'qualitative monitoring' through sentinel sites for iterative participatory investigation into the evolving local socio-cultural, behavioural and practical issues that impact malaria prevention and treatment; identifying and mobilising social networks and their leaders through consensus building processes; intersectoral collaboration; integration of malaria interventions with activities addressing other community health and disease priorities; and targeted implementation of locally-appropriate, multi-level, pulsed media campaigns that sustain motivation for community participation in malaria elimination.

## Competing interests

The authors declare that they have no competing interests.

## Authors' contributions

JA, HT, LF, GT, IR & AV participated in the conception of study design. Training of field researchers in qualitative methods and logistics was carried out by LF, JA & HT. The field research activities were supported by AT, JA & HT. Data analysis and manuscript drafting was carried out by JA with support and contributions from AV, LF, MW & AT. All authors have read and approved the final manuscript.
